# Label-Free Virtual HER2 Immunohistochemical Staining of Breast Tissue using Deep Learning

**DOI:** 10.34133/2022/9786242

**Published:** 2022-10-25

**Authors:** Bijie Bai, Hongda Wang, Yuzhu Li, Kevin de Haan, Francesco Colonnese, Yujie Wan, Jingyi Zuo, Ngan B. Doan, Xiaoran Zhang, Yijie Zhang, Jingxi Li, Xilin Yang, Wenjie Dong, Morgan Angus Darrow, Elham Kamangar, Han Sung Lee, Yair Rivenson, Aydogan Ozcan

**Affiliations:** ^1^Electrical and Computer Engineering Department, University of California, Los Angeles, CA 90095, USA; ^2^Bioengineering Department, University of California, Los Angeles 90095, USA; ^3^California NanoSystems Institute (CNSI), University of California, Los Angeles, CA, USA; ^4^Computer Science Department, University of California, Los Angeles, CA, USA; ^5^Physics and Astronomy Department, University of California, Los Angeles, CA 90095, USA; ^6^Translational Pathology Core Laboratory, University of California, Los Angeles, CA 90095, USA; ^7^Statistics Department, University of California, Los Angeles, CA 90095, USA; ^8^Department of Pathology and Laboratory Medicine, University of California at Davis, Sacramento, CA 95817, USA; ^9^Department of Surgery, University of California, Los Angeles, CA 90095, USA

## Abstract

The immunohistochemical (IHC) staining of the human epidermal growth factor receptor 2 (HER2) biomarker is widely practiced in breast tissue analysis, preclinical studies, and diagnostic decisions, guiding cancer treatment and investigation of pathogenesis. HER2 staining demands laborious tissue treatment and chemical processing performed by a histotechnologist, which typically takes one day to prepare in a laboratory, increasing analysis time and associated costs. Here, we describe a deep learning-based virtual HER2 IHC staining method using a conditional generative adversarial network that is trained to rapidly transform autofluorescence microscopic images of unlabeled/label-free breast tissue sections into bright-field equivalent microscopic images, matching the standard HER2 IHC staining that is chemically performed on the same tissue sections. The efficacy of this virtual HER2 staining framework was demonstrated by quantitative analysis, in which three board-certified breast pathologists blindly graded the HER2 scores of virtually stained and immunohistochemically stained HER2 whole slide images (WSIs) to reveal that the HER2 scores determined by inspecting virtual IHC images are as accurate as their immunohistochemically stained counterparts. A second quantitative blinded study performed by the same diagnosticians further revealed that the virtually stained HER2 images exhibit a comparable staining quality in the level of nuclear detail, membrane clearness, and absence of staining artifacts with respect to their immunohistochemically stained counterparts. This virtual HER2 staining framework bypasses the costly, laborious, and time-consuming IHC staining procedures in laboratory and can be extended to other types of biomarkers to accelerate the IHC tissue staining used in life sciences and biomedical workflow.

## 1. Introduction

The immunohistochemical (IHC) staining of tissue sections plays a pivotal role in the evaluation process of a broad range of diseases. Since its first implementation in 1941 [1], a great variety of IHC biomarkers have been validated and employed in clinical and research laboratories for characterization of specific cellular events [2], e.g., the nuclear protein Ki-67 associated with cell proliferation [3], the cellular tumor antigen P53 associated with tumor formation [4], and the human epidermal growth factor receptor 2 (HER2) associated with aggressive breast tumor development [5]. Due to its capability of selectively identifying targeted biomarkers, IHC staining of tissue has been established as one of the gold standards for tissue analysis and diagnostic decisions, guiding disease treatment and investigation of pathogenesis [6–8].

Though widely used, the IHC staining of tissue still requires a dedicated laboratory infrastructure and skilled operators (histotechnologists) to perform laborious tissue preparation steps and is therefore time-consuming and costly. Recent years have seen rapid advances in deep learning-based virtual staining techniques, providing promising alternatives to the traditional histochemical staining workflow by computationally staining the microscopic images captured from label-free thin tissue sections, bypassing the laborious and costly chemical staining process. Such label-free virtual staining techniques have been demonstrated using autofluorescence imaging [9, 10], quantitative phase imaging [11], and light scattering imaging [12], among others [13–15], and have successfully created multiple types of histochemical stains, e.g., hematoxylin and eosin (H&E) [9–14], Masson’s trichrome [9–11], and Jones silver stains [9–11]. These previous works did not perform any virtual IHC staining and mainly focused on the generation of structural tissue staining, which enhances the contrast of specific morphological features in tissue sections. In a related line of research, deep learning has also enabled the prediction of biomarker status (e.g., Ki-67 [16] and *β*-amyloid [17]) and tumor prognostic from H&E-stained microphotographs of various lesions including hepatocellular carcinoma [18], breast cancer [19–23], bladder cancer [24], thyroid cancer [25, 26], melanoma [27], and neuropathologic diseases [17]. These studies highlight a possible correlation between the presence of specific biomarkers and morphological microscopic changes in the tissue; however, they do not provide an alternative to IHC stained tissue images that reveal subcellular biomarker information for pathologists’ diagnostic inspection for inter- and intracellular signatures such as cytoplasmic and nuclear details [28].

**Figure 1:**
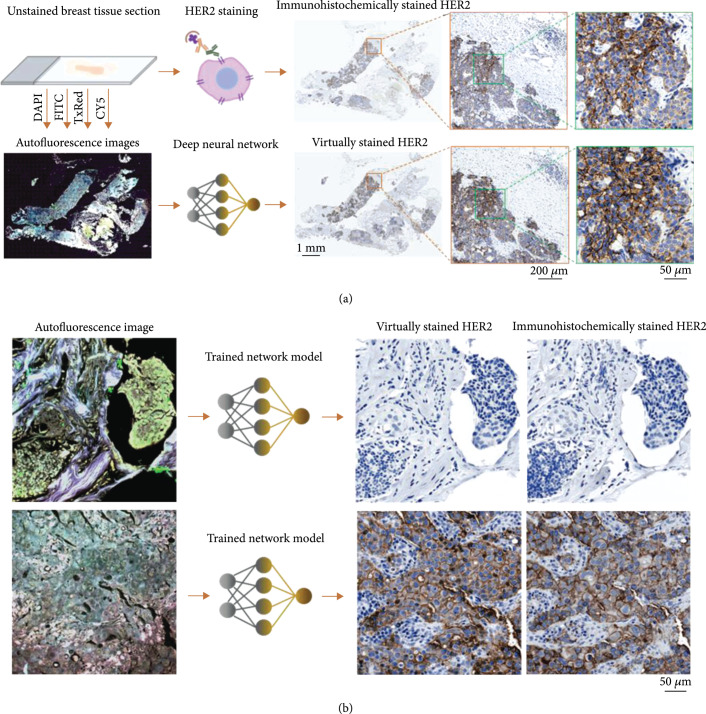
Virtual HER2 staining of unlabeled tissue sections via deep learning. (a) The standard immunohistochemical (IHC) HER2 staining (top) relies on tedious and costly tissue processing performed by histotechnologists, which typically takes ~1 day. A pretrained deep neural network enables virtual HER2 staining of unlabeled tissue sections (bottom). (b) Virtual HER2 staining transforms autofluorescence images of unlabeled tissues sections into bright-field equivalent images that match the images of standard IHC HER2 staining.

Here, we present a deep learning-based label-free virtual IHC staining method (Figure 1), which transforms autofluorescence microscopic images of unlabeled tissue sections into bright-field equivalent images, matching the standard IHC stained images of the same tissue samples. In this study, we specifically focused on the IHC staining of HER2, which is an important cell surface receptor protein that is involved in regulating cell growth and differentiation [29, 30]. Assessing the level of HER2 expression in breast tissue, i.e., HER2 status, is routinely practiced based on the HER2 IHC staining of the formalin-fixed, paraffin-embedded (FFPE) tissue sections and helps predict the prognosis of breast cancer and its response to HER2-directed immunotherapies [5, 30–34]. For example, the intracellular and extracellular studies of HER2 have led to the development of pharmacological anti-HER2 agents that benefit the treatment of HER2-positive tumors [35–39]. Further efforts are being made to develop new pharmacological solutions that can counter HER2-directed-drug resistance and improve treatment outcomes in clinical trials [40–43]. With numerous animal models established for preclinical studies and life sciences related research, a deeper understanding of the oncogene, biological functionality, and drug resistance mechanisms of HER2 is being explored [44–48]. In addition to these, HER2 biomarker was also used as an essential tool in developing and testing of novel biomedical imaging [49, 50], statistics [51], and spatial transcriptomics [52] methods.

**Figure 2:**
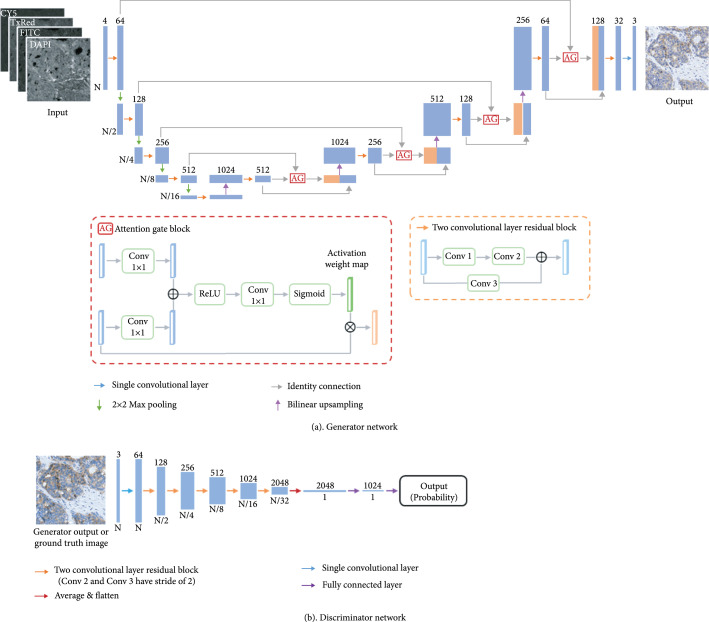
Virtual HER2 staining network. A GAN framework which consists of a generator model and a discriminator model was used to train the virtual HER2 staining network. (a) The generator uses an attention-gated U-net structure to map the label-free autofluorescence images into bright-field equivalent HER2 images. (b) The discriminator is a CNN composed of five successive two-convolutional-layer residual blocks and two fully connected layers (see Methods). Once the network models converge, only the generator model is used to infer the virtual HER2 images, which takes ~12 seconds for 1 mm^2^ of tissue area.

The presented virtual HER2 staining method is based on a deep learning-enabled image-to-image transformation, using a conditional generative adversarial network (GAN), as shown in Figure 2. Once the training phase was completed, two blinded quantitative studies were performed using new breast tissue sections with different HER2 scores to demonstrate the efficacy of our virtual HER2 staining framework. For this purpose, we used the semi-quantitative Dako HercepTest scoring system [53], which involves assessing the percentage of tumor cells that exhibit membranous staining for HER2 along with the intensity of the staining. The results are reported as 0 (negative), 1+ (negative), 2+ (weakly positive/equivocal), and 3+ (positive). In the first study, three board-certified breast pathologists blindly graded the HER2 scores of virtually stained HER2 whole slide images (WSIs) as well as their IHC stained standard counterparts. Our results and the statistical analysis revealed that determining the HER2 status based on our virtual HER2 WSIs is as accurate as standard analysis based on the chemically prepared IHC HER2 slides. In the second study, the same pathologists rated the staining quality of both virtual HER2 and standard IHC HER2 images using different metrics, i.e., nuclear detail, membrane clearness, background staining, and staining artifacts. This study revealed that at least two pathologists out of the three agreed that there is no statistically significant difference between the virtual HER2 staining image quality and the standard IHC HER2 staining image quality in the level of nuclear detail, membrane clearness, and absence of staining artifacts. Additional feature-based quantitative assessments also confirmed the high degree of agreement between the virtually generated HER2 images and their standard IHC-stained counterparts, in terms of both nucleus and membrane stain features.

The presented framework achieved the first demonstration of label-free virtual IHC staining and bypasses the costly, laborious, and time-consuming IHC staining procedures that involve toxic chemical compounds. This virtual HER2 staining technique has the potential to be extended to virtual staining of other biomarkers and may accelerate the IHC-based tissue analysis workflow in life sciences and biomedical applications, while also enhancing the repeatability and standardization of IHC staining.

## 2. Results

### 2.1. Label-Free Virtual HER2 Staining of Breast Tissue

We demonstrated our virtual HER2 staining method by training deep neural network (DNN) models with a dataset of 25 breast tissue sections collected from 19 unique patients, constituting in total 20,910 image patches, each with 1024×1024 pixels. Once a DNN model was trained, it virtually stained the unlabeled tissue sections using their autofluorescence microscopic images captured with DAPI, FITC, TxRed, and Cy5 filter cubes (see Methods section), matching the corresponding bright-field images of the same field-of-views, captured after standard IHC HER2 staining. In the network training and evaluation process, we employed a cross-validation approach. Separate network models were trained with different dataset divisions to generate 12 virtual HER2 WSIs for blind testing, i.e., 3 WSIs at each of the 4 HER2 scores (0, 1+, 2+, and 3+). Each virtual HER2 WSI corresponds to a unique patient that was not used during the network training phase. Note that all the tissue sections were obtained from existing tissue blocks, where the HER2 reference (ground truth) scores were provided by UCLA Translational Pathology Core Laboratory (TPCL) under UCLA IRB 18-001029.

**Figure 3:**
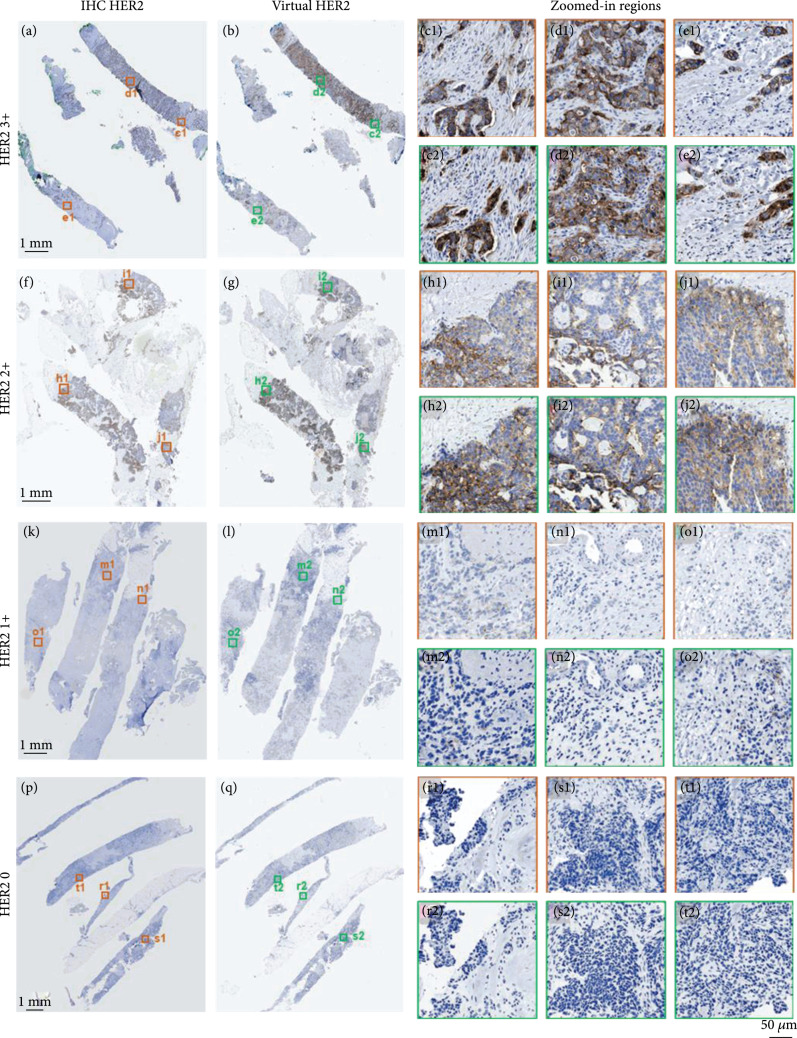
Comparison of virtual and standard IHC HER2 staining of breast tissue sections at different HER2 scores. (a), (f), (k), (p) Bright-field WSIs of standard IHC HER2 stained samples at a HER2 3+, f HER2 2+, k HER2 1+, and p HER2 0. (b), (g), (l), (q) Bright-field WSIs generated by virtual staining, corresponding to the same samples as (a), (f), (l), and (p), respectively. (c1)-(e1), (c2)-(e2) Zoomed-in regions of interest from (a) and (b) at a HER2 score of 3+. (h1)-(j1), (h2)-(j2) Zoomed-in regions of interest from (f) and (g) at a HER2 score of 2+. (m1)-(o1), (m2)-(o2) Zoomed-in regions of interest from (k) and (l) at a HER2 score of 1+. (r1)-(t1), (h2)-(t2) Zoomed-in regions of interest from (p) and (q) at a HER2 score of 0.

Figure 3 summarizes the comparison of the virtual HER2 images inferred by our DNN models against their corresponding IHC HER2 images captured from the same tissue sections after standard IHC staining. Both the WSIs and the zoomed-in regions show a high degree of agreement between virtual staining and standard IHC staining. These results indicate that a well-trained virtual staining network can reliably transform the autofluorescence images of unlabeled breast tissue sections into the bright-field equivalent, virtual HER2 images, which match their IHC HER2 stained counterparts, across all the HER2 statuses, 0, 1+, 2+, and 3+. Upon close examination, our board-certified pathologists confirmed that the comparison between the IHC and virtual HER2 images showed equivalent staining with no significant perceptible differences in intracellular features such as membrane clarity or nuclear details. In particular, the virtual staining network clearly produced the expected intensity and distribution of membranous HER2 staining (or lack thereof) in tumor cells. In HER2 positive (3+, Figures 3(a)–3(e)) breast cancers, both virtually stained and IHC stained images showed strong complete membranous staining in >10% of tumor cells, as well as dim cytoplasmic staining in tumor cells. None of the stromal and inflammatory cells showed false-positive staining, and the nuclear details of the tumor cells were comparable in both panels. In equivocal (2+, Figures 3(f)–3(j)) tumors, virtual images showed weak to moderate membranous staining in >10% of tumor cells, providing the same amount of membranous staining of tumor cells in corresponding areas. HER2 negative (1+, Figures 3(k)–3(o)) tumors showed faint membranous staining in 10% or more of tumor cells. None of the stromal and inflammatory cells showed faint staining. HER2 negative (0, Figures 3(p)–3(t)) tumor showed no staining in the tumor cells.

### 2.2. Blind Evaluation and Quantification of Virtual HER2 Staining

**Figure 4:**
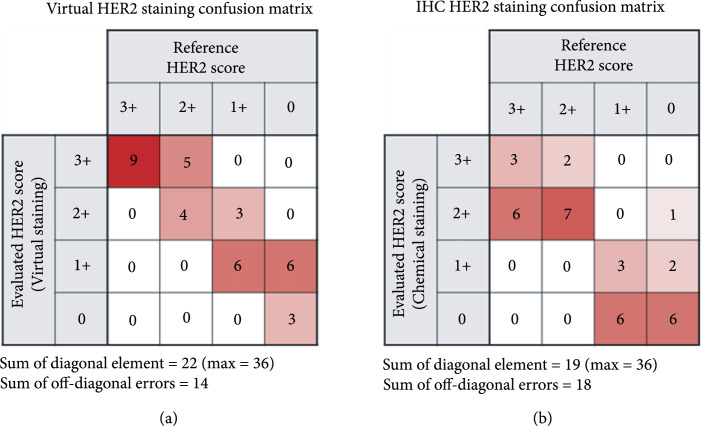
Confusion matrices of HER2 scores. Each element in the matrices represents the number of WSIs with their HER2 scores evaluated by board-certified pathologists (rows) based on: (a) virtual HER2 staining or (b) standard IHC HER2 staining, compared to the reference (ground truth) HER2 scores provided by UCLA TPCL (columns).

Next, we evaluated the efficacy of the presented virtual HER2 staining framework with a quantitative blinded study in which the 12 virtual HER2 WSIs and their corresponding standard IHC HER2 WSIs were mixed and presented to three board-certified breast pathologists who graded the HER2 score (i.e., 3+, 2+, 1+, or 0) for each WSI without knowing if the image was from a virtual stain or standard IHC stain. Random image shuffling, rotation, and flipping were applied to the WSIs to promote blindness in evaluations. The HER2 scores of the virtual and the standard IHC WSIs that were blindly graded by the three pathologists are summarized in Figure 4 and compared to their reference, ground truth scores provided by UCLA TPCL. The confusion matrices of virtual HER2 WSIs (Figure 4(a)) and IHC HER2 WSIs (Figure 4(b)), each corresponding to N=36 evaluations, reveal that our virtual HER2 staining approach achieved a similar level of accuracy for HER2 status assessment as the standard IHC staining. Close examination of these confusion matrices reveals that the sum of the diagonal elements of the virtual HER2-based evaluations (22) is higher than that of the IHC HER2 (19), showing that more cases were correctly scored based on virtual HER2 WSIs compared to those based on standard IHC HER2 WSIs. Furthermore, the sum of the absolute off-diagonal errors of virtual HER2-based evaluations (14) is smaller than that of the standard IHC HER2 (18). Based on the same confusion matrices shown in Figure 4, a Chi-square test was performed to compare the degree of agreement between virtual staining and standard IHC staining methods in HER2 scoring. The test results indicate that there is no statistically significant difference between the two methods (P=0.4752, see Supplementary Table 1).

**Figure 5:**
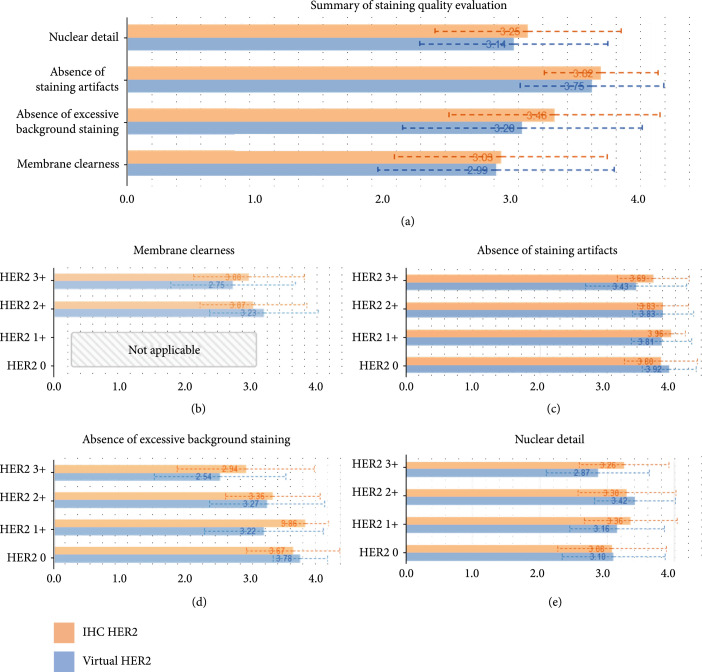
Comparisons of image quality of virtual HER2 and standard IHC HER2 staining. (a) Quality scores of virtual HER2 and standard IHC HER2 images calculated based on 4 different feature metrics: nuclear details, absence of staining artifacts, absence of excessive background staining, and membrane clearness. Each value was averaged over all the image patches and pathologists. (b)–(e) Detailed comparisons of quality scores under each feature metric at different HER2 scores. The grade scale applied for each metric is 1 to 4: 4 for perfect, 3 for very good, 2 for acceptable, and 1 for unacceptable. The standard deviations are plotted by dashed lines.

In addition to evaluating the efficacy of virtual staining in HER2 scoring, we also quantitatively evaluated the staining quality of the virtual HER2 images and compared them to the standard IHC HER2 images. In this blinded study, we randomly extracted 10 regions-of-interest (ROIs) from each of the 12 virtual HER2 WSIs and 10 ROIs at the same locations from each of their corresponding IHC HER2 WSIs, building a test set of 240 image patches. Each image patch has 8000×8000 pixels (1.3×1.3 mm^2^), which was also randomly shuffled, rotated, and flipped before being reviewed by the same three pathologists. These pathologists were asked to grade the image quality of each ROI based on four predesignated feature metrics for HER2 staining: membrane clearness, nuclear detail, absence of excessive background staining, and absence of staining artifacts (Figure 5). The grade scale for each metric is from 1 to 4, with 4 representing perfect, 3 representing very good, 2 representing acceptable, and 1 representing unacceptable. Figure 5(a) summarizes the staining quality scores of virtual HER2 and standard IHC HER2 images based on our predefined feature metrics, which were averaged over all image patches and pathologists. Figures 5(b)–5(e) further compare the average quality scores at each of the 4 HER2 statuses under each feature metric. In Figure 5(b), the membrane clearness scores of HER2 negative ROIs are noted as “not applicable” since there is no staining of the cell membrane in HER2 negative samples. It is important to emphasize that the standard IHC HER2 images had an advantage in these comparisons because they were preselected: A significant percentage of the standard IHC HER2 tissue slides suffered from unacceptable staining quality issues (see Discussion and Supplementary Figure 1), and therefore they were excluded from our comparative studies in the first place. Nevertheless, the quality scores of virtual and standard IHC HER2 staining are very close to each other and fall within their standard deviations (dashed lines in Figure 5). We also performed one-sided t-tests on each feature metric evaluated by board-certified pathologists to determine whether standard IHC HER2 images are statistically significantly better than the virtual HER2 images in staining quality. The t-test results showed that only for the metric of “absence of excessive background staining,” two of the three pathologists reported a statistically significant improvement in the quality of the standard IHC staining compared to the virtual staining. For the rest of the feature metrics (i.e., nuclear details, membrane clearness, and staining artifacts), at least two of the three pathologists reported that the staining quality of the IHC HER2 images is not statistically significantly better than their virtual HER2 counterparts (Supplementary Table 2). Also note that the virtually stained HER2 images did not mislead the diagnosis at the whole slide level as also analyzed using the confusion matrices shown in Figure 4 and the Chi-square test reported in Supplementary Table 1.

Besides rating the staining quality of each ROI, the pathologists also graded a HER2 score for each ROI, the results of which are reported in Supplementary Figure 2. Each histogram in Supplementary Figure 2a summarizes the HER2 scores of the 10 ROIs extracted from each WSI evaluated by 3 pathologists (i.e., N=30 evaluations). The reference (ground truth) HER2 scores of the corresponding WSIs are plotted as gray dashed lines. This analysis reveals that, for the majority of the patients, there is no discrepancy between HER2 scores evaluated from virtually generated ROIs and standard IHC stained ROIs. For the cases where there is a disagreement (e.g., Patients #5 and #11), the histograms of the virtual HER2 scores were centered closer to the reference HER2 scores (dashed lines) compared to the histograms of the standard IHC-based HER2 scores. It is important to also note that grading the HER2 scores from subsampled ROIs vs. from the WSI can yield different results due to the inhomogeneous nature of the tissue sections.

### 2.3. Feature-Based Quantitative Assessment of Virtual HER2 Staining

In addition to the pathologists’ blind assessments of the virtual staining efficacy and the image quality, we further carried out a feature-based quantitative analysis of the virtually generated HER2 images compared to their IHC-stained counterparts. In this analysis, 8194 unique test image patches (each with a size of 1024×1024 pixels) were blindly selected for virtual staining. Due to the different staining features of each different HER2 status, these blind testing images were divided into two subsets for quantitative evaluation: one subset containing the images from HER2 0 and HER2 1+, N=4142, and the other containing the images from HER2 2+ and HER2 3+, N=4052. For each virtually stained HER2 image and its corresponding IHC HER2 image (ground truth), four feature-based quantitative evaluation metrics (specifically designed for HER2) were calculated based on the segmentation of nucleus stain and membrane stain (see the Methods section). These four feature-based evaluation metrics included the number of nuclei and the average nucleus area (in number of pixels) for quantifying the nucleus stain in each image as well as the area under the characteristic curve and the membrane region connectedness [54, 55] for quantifying the membrane stain in each image (refer to the Methods section for details).

**Figure 6:**
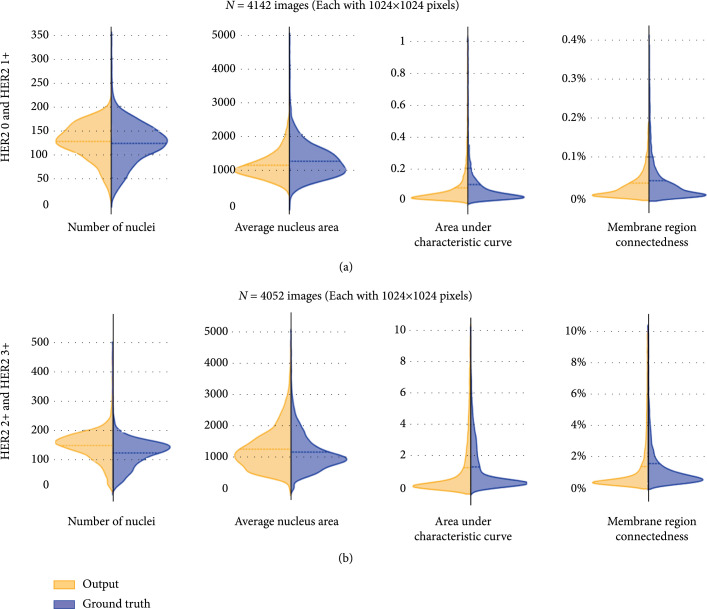
Feature-based quantitative assessment of virtually stained HER2 images and standard IHC HER2 images. (a) Virtual HER2 features and standard IHC HER2 features, quantitatively compared for HER2 negative cases (N=4,142 images) based on four different metrics. (b) Virtual HER2 features and standard IHC HER2 features, quantitatively compared for HER2 positive cases (N=4,052 images) based on the same metrics.

These feature-based quantitative evaluation results for the virtual HER2 images compared against their standard IHC counterparts are shown in Figure 6. This analysis demonstrated that the virtual HER2 staining feature metrics exhibit similar distributions and closely matching average values (dashed lines) compared to their standard IHC counterparts, in terms of both the nucleus and the membrane stains. By comparing the evaluation results of the HER2 positive group (2+ and 3+) against the HER2 negative group (0 and 1+), we observe similar distributions of nucleus features (i.e., the number of nuclei and average nucleus area) and higher levels of membrane stain, which correlates well with the higher HER2 scores as expected.

## 3. Discussion

We demonstrated a deep learning-enabled label-free virtual IHC staining method. By training a DNN model, our method generated virtual HER2 images from the autofluorescence images of unlabeled tissue sections, matching the bright-field images captured after standard IHC staining. Compared to chemically performing the IHC staining, our virtual HER2 staining method is rapid and simple to operate. The conventional IHC HER2 staining involves laborious sample treatment steps demanding a histotechnologist’s periodic monitoring (see Supplementary Note 1), and this whole process typically takes one day before the slides can be reviewed by diagnosticians. In contrast, the presented virtual HER2 staining method bypasses these laborious and costly steps and generates the bright-field equivalent HER2 images computationally using the autofluorescence images captured from label-free tissue sections. After the training is complete (which is a one-time effort), the entire inference process using a virtual staining network only takes ~12 seconds for 1 mm^2^ of tissue using a consumer-grade computer, which can be further improved by using faster hardware acceleration units.

Another advantage of the presented method is its capability of generating highly consistent and repeatable staining results, minimizing the staining variations that are commonly observed in standard IHC staining. The IHC HER2 staining procedure is delicate and laborious as it requires accurate control of time, temperature, and concentrations of the reagents at each tissue treatment step; in fact, it often fails to generate satisfactory stains. In our study, ~30% of the sample slides were discarded because of unsuccessful standard IHC staining and/or severe tissue damage even though the IHC staining was performed by accredited pathology labs. Supplementary Figure 1 shows two examples of the standard IHC staining failures we experienced, including complete tissue damage and false negative staining that failed to reflect the correct HER2 score. In contrast, our computational virtual staining approach does not rely on the chemical processing of the tissue and generates reproducible results, which is important for the standardization of the HER2 interpretation by eliminating commonly experienced staining variations and artifacts.

Since the autofluorescence input images of tissue slices were captured with standard filter sets installed on a conventional fluorescence microscope, the presented approach is ready to be implemented on existing fluorescence microscopes without hardware modifications or customized optical components. Our results showed that the combination of the four commonly used fluorescence filters (DAPI, FITC, TxRed, and Cy5) provided a very good baseline for the virtual HER2 staining performance. As an ablation study, we also quantitatively compared virtual staining networks that are trained with different autofluorescence input channels by calculating peak signal-to-noise ratio (PSNR) and structural similarity index (SSIM) [56] between the network output and ground truth images (see Supplementary Figure 3). Since the staining of the cell membrane is an important assessment factor in HER2 status evaluation, we also performed color deconvolution [57] to split out the membrane stain channel (i.e., diaminobenzidine (DAB) stain) followed by calculating and comparing the SSIM scores (Supplementary Figure 4). These analyses revealed that the performance of the virtual staining network partially degraded with decreasing number of input autofluorescence channels, motivating the use of DAPI, FITC, TxRed, and Cy5 altogether (Supplementary Figure 3b).

The advantages of using the attention-gated GAN structure for virtual HER2 staining are illustrated by an additional comparative study, in which we trained and blindly tested four different network architectures including (1) the attention-gated GAN structure used in this work, (2) the same structure as ours with the residual connections removed, (3) the same structure as ours with the attention-gated blocks removed, and (4) an unsupervised cycleGAN [58, 59] framework. The training/validation/testing datasets and the training epochs were kept the same for all the four networks. After their training, we quantitatively compared these networks by calculating the PSNR, SSIM, and SSIM of the membrane stain (SSIM_DAB_) between the network output and the ground truth images (see Supplementary Figure 5). Both the visual and numerical comparisons revealed that the attention-gated GAN used in this work is the only network architecture that could provide consistently superior and accurate virtual staining results at various HER2 expression levels, while the other network architectures made some catastrophic staining errors in one or more testing FOVs, making them unacceptable for consistent inference across all HER2 statuses. In Supplementary Figures 6 and 7, we further compared the color distributions (see the Methods section) of the output images generated by these different network architectures against the corresponding ground truth images, including FOVs with strong HER2 expression (Supplementary Figure 6) and FOVs with weak HER2 expression (Supplementary Figure 7). These additional comparisons showed that the color histograms of the output images generated by our framework match with the standard IHC ground truth much more closely for both the membrane and nucleus stain channels, which again illustrates the advantages of using the attention-gated GAN architecture reported in this work.

The success of our virtual HER2 staining method relies on the processing of the complex spatial-spectral information that is encoded in the autofluorescence images of label-free tissue using convolutional neural networks. The presented virtual staining method can potentially be expanded to a wide range of other IHC stains. Though our virtual HER2 staining framework was demonstrated based on autofluorescence imaging of unlabeled tissue sections, other label-free microscopy modalities may also be utilized for this task, such as holography [11], fluorescence lifetime imaging [60, 61], and Raman microscopy [62]. In addition to generalizing to other types of IHC stains in the assessment of various biomarkers, this method can be further adapted to nonfixed fresh tissue samples or frozen sections, which can potentially provide real-time virtual IHC images for intraoperative consultation during surgical operations.

To the best of our knowledge, our results (placed in arXiv [63] on December 8, 2021) constitute the first demonstration of label-free virtual IHC staining, and we believe that this framework opens up new avenues for various applications in life sciences and biomedical diagnostics and can potentially transform the traditional IHC staining workflow.

## 4. Methods

### 4.1. Sample Preparation and Standard IHC Staining

The unlabeled breast tissue blocks were provided by the UCLA TPCL under UCLA IRB 18-001029 and were cut into 4 *μ*m thin sections. The FFPE thin sections were then deparaffinized and covered with glass coverslips. After acquiring the autofluorescence microscopic images, the unlabeled tissue sections were sent to accredited pathology labs for standard IHC HER2 staining, which was performed by UCLA TPCL and the Department of Anatomic Pathology of Cedars-Sinai Medical Center in Los Angeles, USA. The IHC HER2 staining protocol provided by UCLA TPCL is described in Supplementary Note 1.

### 4.2. Image Data Acquisition

The autofluorescence images of the unlabeled tissue sections were captured using a standard fluorescence microscope (IX-83, Olympus) with a × 40/0.95NA (UPLSAPO, Olympus) objective lens. Four fluorescent filter cubes, including DAPI (Semrock DAPI-5060C-OFX, EX 377/50 nm, EM 447/60 nm), FITC (Semrock FITC-2024B-OFX, EX 485/20 nm, EM 522/24 nm), TxRed (Semrock TXRED-4040C-OFX, EX 562/40 nm, EM 624/40 nm), and Cy5 (Semrock CY5-4040C-OFX, EX 628/40 nm, EM 692/40 nm) were used to capture the autofluorescence images at different excitation-emission wavelengths. Each autofluorescence image was captured with a scientific complementary metal-oxide-semiconductor (sCMOS) image sensor (ORCA-flash4.0 V2, Hamamatsu Photonics) with an exposure time of 150 ms, 500 ms, 500 ms, and 1000 ms for DAPI, FITC, TxRed, and Cy5 filters, respectively. The image acquisition process was controlled by *μ*Manager (version 1.4) microscope automation software [64]. After the standard IHC HER2 staining is complete, the bright-field WSIs were acquired using a slide scanner microscope (AxioScan Z1, Zeiss) with a × 20/0.8NA objective lens (Plan-Apo).

### 4.3. Image Preprocessing and Registration

The matching of the autofluorescence (network input) and the bright-field IHC HER2 (network ground truth) image pairs is critical for the successful training of an image-to-image transformation network. The image processing workflow for preparing the training dataset for our virtual HER2 staining network is described in Supplementary Figure 8, which was implemented in MATLAB (MathWorks). First, the autofluorescence images (before the IHC staining) and the whole-slide bright-field images (after the IHC staining) of the same tissue sections were stitched into WSIs (Supplementary Figure 8(a)) and globally co-registered by detecting and matching the speeded up robust features (SURF) points [65] (Supplementary Figure 8(b)). Then, these coarsely matched autofluorescence and bright-field WSIs were cropped into pairs of image tiles of 1024×1024 pixels (Supplementary Figure 8(c)). These image pairs were not accurately matched at the pixel level due to optical aberrations and morphological changes of the tissue structure during the standard (laborious) IHC staining procedures. In order to calculate the transformation between the autofluorescence image and its bright-field counterpart using a correlation-based elastic registration algorithm [66], a registration model [9] needs to be trained to match the style of the autofluorescence images to the style of the bright-field images (Supplementary Figure 8d). This registration network used the same architecture as our virtual staining network. Following the image style transformation using the registration network (Supplementary Figure 8(e)), the pyramid elastic image registration algorithm [66, 67] was performed to hierarchically match the local features of the sub-image blocks and calculate the transformation maps. The transformation maps were then applied to correct for the local wrappings of the ground truth images (Supplementary Figure 8(f)) which were then better matched to their autofluorescence counterparts. This training-registration process (Supplementary Figures 8(d)-8(f)) was repeated 3-5 times until the autofluorescence input and the bright-field ground truth image patches were accurately matched at the single pixel-level (Supplementary Figure 8(g)). At last, a manual data cleaning process was performed to remove image pairs with artifacts such as tissue-tearing (during the standard chemical staining process) or defocusing (during the imaging process).

### 4.4. Virtual HER2 Staining Network Architecture and Training Schedule

In this work, a GAN-based network model [68] was employed to perform the transformation from the 4-channel label-free autofluorescence images (DAPI, FITC, TxRed, and Cy5) to the corresponding bright-field virtual HER2 images, as shown in Figure 2. This GAN framework includes (1) a generator network that creates virtually stained HER2 images by learning the statistical transformation between the input autofluorescence images and the corresponding bright-field IHC stained HER2 images (ground truth) and (2) a discriminator network that learns to discriminate the virtual HER2 images created by the generator from the actual IHC stained HER2 images. The generator and the discriminator were alternatively optimized and simultaneously improved through this competitive training process. Specifically, the generator (*G*) and discriminator (*D*) networks were optimized to minimize the following loss functions:

(1)lgenerator=α×L1Itarget,GIinput−λ×log1+SSIMItarget,GIinput/2+γ×BCEDGIinput,1,ldiscriminator=BCEDGIinput,0+BCEDItarget,1,

where $G\left(∙\right)\;$ represents the generator inference; $D\left(∙\right)\;$ represents the probability of being a real, actually-stained IHC image predicted by the discriminator; $I_{\text{input}}$ denotes the input label-free autofluorescence images; and $I_{\text{target}}$ denotes the ground truth, standard IHC stained image. The coefficients (α,λ,γ) in $l_{\text{generator}}$ were empirically set as (10, 0.2, and 0.5) to balance the pixel-wise smooth $L_{1}$ error [69] of the generator output with respect to its ground truth, SSIM loss [56] of the generator output, and the binary cross-entropy (BCE) loss of the discriminator predictions of the output image. Compared to using the mean squared error (MSE) loss, the smooth $L_{1}$ loss is a robust estimator that prevents exploding gradients by using MSE around zero and mean absolute error (MAE) in other parts [70]. Specifically, smooth $L_{1}$ loss between two images A and B is defined as

(2)L1A,B=1M×N∑m,nAm,n−Bm,n<β0.5×Am,n−Bm,n2β+∑m,nAm,n−Bm,n≥βAm,n−Bm,n−0.5β,

where m and n are the pixel indices, the M×N represents the total number of pixels in each image, and β was set to 1 in our case.

The SSIM of two images   is defined as [56]

(3)SSIMA,B=2μAμB+c12σAB+c2μA2+μB2+c1σA2+σB2+c2,

where $\mu_{A}$ and $\mu_{B}$ are the mean values of the images A and B, $\sigma_{A}^{2}$ and $\sigma_{B}^{2}$ are the variance of images A and B, and $\sigma_{AB}$ is the covariance between images A and B. $c_{1}$ and $c_{2}$ were set to be $0.01^{2}$ and $0.03^{2}$, respectively [56].

The BCE with logits loss used in our network is defined as

(4)BCEp,q=−q×logsigmoidp+1−q×log1−sigmoidp,

where p represents the discriminator predictions and q represents the actual labels (0 or 1).

As shown in Figure 2(a), the generator network was built following the attention U-Net architecture [71] with 4 resolution levels, which can map the label-free autofluorescence images into the HER2 stained images by learning the transformations of spatial features at different spatial scales, catching both the high-resolution local features at shallower levels and the larger scale global context at deeper levels. Our attention U-Net structure is composed of a downsampling path and an upsampling path that are symmetric to each other. The downsampling path contains four downsampling convolutional blocks, each consisting of a two-convolutional-layer residual block, followed by a leaky rectified linear unit [72] (Leaky ReLU) with a slope of 0.1, and a 2×2 max pooling operation with a stride size of 2 for downsampling. The two-convolutional-layer residual blocks contain two consecutive convolutional layers with a kernel size of 3×3 and a convolutional residual path [73] connecting the in and out tensors of the two convolutional layers. The numbers of the input channels and the output channels at each level of the downsampling path were set to 4, 64, 128, and 256 and 64, 128, 256, and 512, respectively.

Symmetrically, the upsampling path contains four upsampling convolutional blocks with the same design as the downsampling convolutional blocks, except that the 2× downsampling operation was replaced by a 2× bilinear upsampling operation. The input of each upsampling block is the concatenation of the output tensor from the previous block with the corresponding feature maps at the matched level of the downsampling path passing through the attention gated connection. An attention gate consists of three convolutional layers and a sigmoid operation, which outputs an activation weight map highlighting the salient spatial features [71]. The numbers of the input channels and the output channels at each level of the upsampling path were 1024, 1024, 512, and 256 and 1024, 512, 256, and 128, respectively. Following the upsampling path, a two-convolutional layer residual block together with another single convolutional layer reduces the number of channels to 3, matching that of our ground truth images (i.e., 3-channel RGB images). Additionally, a two-convolutional-layer center block was utilized to connect and match the dimensions of the downsampling path and the upsampling path.

The structure of the discriminator network is illustrated in Figure 2(b). An initial block containing one convolutional layer followed by a Leaky ReLU operation first transformed the 3-channel generator output or ground truth image to a 64-channel tensor. Then, five successive two-convolutional-layer residual blocks were added to perform 2× downsampling and expand the channel numbers of each input tensor. The 2× downsampling was enabled by setting the stride size of the second convolutional layer in each block as 2. After passing through the five blocks, the output tensor was averaged and flattened to a one-dimensional vector, which was then fed into two fully connected layers to obtain the probability of the input image being the standard IHC-stained image.

The full image dataset contains 25 WSIs from 19 unique patients, making a set of 20,910 image patches, each with a size of 1024×1024 pixels. For the training of each virtual staining model used in our cross-validation studies, the dataset was divided as follows: (1) Test set: images from the WSIs of 1-2 unique patients (~10%, not overlapped with training or validation patients); after splitting out the test set, the remaining WSIs were further divided to the (2) validation set: images from 2 of the WSIs (~10%), and (3) training set: images from the remaining WSIs (~80%). The network models were optimized using image patches of 256×256 pixels, which were randomly cropped from the images of 1024×1024 pixels in the training dataset. An Adam optimizer with weight decay [74] was used to update the learnable parameters at a learning rate of $1\times 10^{-4}$ for the generator network and $1\times 10^{-5}$ for the discriminator network, with a batch size of 28. The generator/discriminator update frequency was set to 2 : 1. Finally, the best model was selected based on the best MSE loss, assisted with the visual assessment of the validation images. The networks converged after ~120 hours of training.

### 4.5. Implementation Details

The image preprocessing was implemented in MATLAB using version R2018b (MathWorks). The virtual staining network was implemented using Python version 3.9.0 and Pytorch version 1.9.0. The training was performed on a desktop computer with an Intel Xeon W-2265 central processing unit (CPU), 64 GB random-access memory (RAM), and an Nvidia GeForce GTX 3090 graphics processing unit (GPU).

### 4.6. Pathologists’ Blind Evaluation of HER2 Images

For the evaluation of WSIs, 24 high-resolution WSIs were randomly shuffled, rotated, flipped, and uploaded to an online image viewing platform that was shared with three board-certified pathologists to blindly evaluate and score the HER2 status of each WSI using the Dako HercepTest scoring system [53]. For the evaluation of sub-ROI images, the 240 image patches were randomly shuffled, rotated, flipped, and uploaded to an online image sharing platform GIGAmacro (https://www.gigamacro.com/). These 240 image patches used for staining quality evaluation can be accessed at https://viewer.gigamacro.com/collections/u08mwIpUDAwfR1vQ?sb=date&sd=asc.

The pathologists’ blinded assessments are provided in Supplementary Data 1.

### 4.7. Statistical Analysis

A Chi-square test (two-sided) was performed to compare the agreement of the HER2 scores evaluated based on the virtual staining and the standard IHC staining. Paired t-tests (one-sided) were used to compare the image quality of virtual staining vs. standard IHC staining. We first calculated the differences between the scores of the virtual and IHC image patches cropped from the same positions, i.e., subtracted the score of each IHC stained image from the score of the corresponding virtually stained image. Then, one-sided t-tests were performed to compare the differences with 0, by each feature metric and each pathologist (see the Supplementary Information). For all tests, a P value of ≤0.05 was considered statistically significant. All the analyses were performed using SAS v9.4 (The SAS Institute, Cary, NC).

### 4.8. Numerical Evaluation of HER2 Images

For the feature-based quantitative assessment of HER2 images (reported in Figure 6), a color deconvolution [57] was performed to separate the nucleus stain channel (i.e., hematoxylin stain) and the membrane stain channel (i.e., diaminobenzidine stain, DAB), as shown in Supplementary Figure 9. The nucleus segmentation map was obtained using the Otsu’s thresholding method [75] followed by morphological operations (e.g., image erosion and image dilation) on the hematoxylin channel. Based on the binary nucleus segmentation map, the number of nuclei and the average nucleus area were extracted by counting the number of connected regions and measuring the average region area. For the evaluation of the membrane stain, the separated DAB image channel was first transformed into the HSV color space. Then, the segmentation map of the membrane stain was obtained by applying a threshold (s) to the saturation channel. By gradually increasing the threshold value (s) from 0.1 to 0.5 with a step size of 0.02, the ratio of the total segmented membrane stain area to the entire image FOV (i.e., 1024×1024 pixels) was calculated, creating the characteristic curve [54] (Supplementary Figure 9). The area under the characteristic curve can be accordingly extracted, providing a robust metric for evaluating HER2 expression levels. By setting the threshold value (s) to 0.25, the ratio of the largest connected component in the membrane segmentation map to the entire image FOV was also extracted as the membrane region connectedness [55].

For the characterization of the color distribution reported in Supplementary Figures 6-7, the nucleus stain channel and the membrane stain channel were split using the same color deconvolution method [57] as in Supplementary Figure 9. For each stain channel, the histogram of all the normalized pixel values was created and followed by a nonparametric kernel-smoothing to fit the distribution profile [76]. y-axes (i.e., the frequency) of the color histograms shown in Supplementary Figures 6-7 were normalized by the total pixel counts.

## Data Availability

Data supporting the results demonstrated by this study are available within the main text and the Supplementary Information. The full set of images used for the HER2 status and stain quality assessment studies can be found in the Supplementary Data 1 file and at: https://viewer.gigamacro.com/collections/u08mwIpUDAwfR1vQ?sb=date&sd=asc. The full pathologist reports can be found in the Supplementary Data 1 file. The full statistical analysis report can be found in Supplementary Data 2 file. Raw WSIs corresponding to patient specimens were obtained under UCLA IRB 18-001029 from the UCLA Health private database for the current study and therefore cannot be made publicly available.

## Additional Points

*Code availability*. All the deep-learning models used in this work employ standard libraries and scripts that are publicly available in Pytorch. The codes used in this manuscript can be accessed through GitHub: https://github.com/baibijie/HER2-virtual-staining.

## Supplementary Materials

Supplementary Materials The Supplementary Materials include Supplementary Figures 1-9, Supplementary Tables 1-2 and Supplementary Note 1 (IHC HER2 staining protocol).
