# Supplementary material for: Label-Free Virtual HER2 Immunohistochemical Staining of Breast Tissue using Deep Learning
Source: BME Front. 2022 Oct 25;2022:9786242. doi: 10.34133/2022/9786242 (PMC10521710; doi:10.34133/2022/9786242)
Supplement: Supplementary Materials — The Supplementary Materials include Supplementary Figures 1-9, Supplementary Tables 1-2 and Supplementary Note 1 (IHC HER2 staining protocol). [file 9786242.f1.zip › Supplementary Data 2 - Statistical analysis report TBS.rtf]

Table of pathologist by target_output	
pathologist	target_output	
Frequency
Col Pct	output	target	Total	
Disagree	14
38.89	17
47.22	31
	
Agree	22
61.11	19
52.78	41
	
Total	36	36	72	


Statistics for Table of pathologist by target_output	


Statistic	DF	Value	Prob	
Chi-Square	1	0.5098	0.4752	
Likelihood Ratio Chi-Square	1	0.5105	0.4749	
Continuity Adj. Chi-Square	1	0.2266	0.6341	
Mantel-Haenszel Chi-Square	1	0.5028	0.4783	
Phi Coefficient		-0.0841		
Contingency Coefficient		0.0839		
Cramer's V		-0.0841		


Fisher's Exact Test	
Cell (1,1) Frequency (F)	14	
Left-sided Pr <= F	0.3172	
Right-sided Pr >= F	0.8294	
		
Table Probability (P)	0.1466	
Two-sided Pr <= P	0.6344	

Sample Size = 72	

target_output	N Obs	Variable	N	Mean	Std Dev	Minimum	Maximum	
output	60	Pathologist_1
Pathologist_2
Pathologist_3	60
59
60	3.3333333
3.4406780
2.2166667	0.6552698
0.8763401
0.6911466	2.0000000
1.0000000
1.0000000	4.0000000
4.0000000
4.0000000	
target	60	Pathologist_1
Pathologist_2
Pathologist_3	60
59
60	3.2333333
3.5593220
2.3166667	0.5325559
0.7256670
0.6762726	2.0000000
1.0000000
1.0000000	4.0000000
4.0000000
4.0000000	

N	Mean	Std Dev	Std Err	Minimum	Maximum	
60	0.1000	0.7059	0.0911	-2.0000	2.0000	


Mean	95% CL Mean	Std Dev	95% CL Std Dev	
0.1000	-Infty	0.2523	0.7059	0.5984	0.8610	


DF	t Value	Pr < t	
59	1.10	0.8615	


N	Mean	Std Dev	Std Err	Minimum	Maximum	
58	-0.1207	1.0773	0.1415	-3.0000	2.0000	


Mean	95% CL Mean	Std Dev	95% CL Std Dev	
-0.1207	-Infty	0.1158	1.0773	0.9108	1.3190	


DF	t Value	Pr < t	
57	-0.85	0.1986	


N	Mean	Std Dev	Std Err	Minimum	Maximum	
60	-0.1000	1.0528	0.1359	-2.0000	2.0000	


Mean	95% CL Mean	Std Dev	95% CL Std Dev	
-0.1000	-Infty	0.1271	1.0528	0.8924	1.2841	


DF	t Value	Pr < t	
59	-0.74	0.2324	


target_output	N Obs	Variable	N	Mean	Std Dev	Minimum	Maximum	
output	120	Pathologist_1
Pathologist_2
Pathologist_3	120
120
120	3.2000000
3.8250000
2.5833333	0.7735110
0.5291900
1.0090626	2.0000000
1.0000000
1.0000000	4.0000000
4.0000000
4.0000000	
target	120	Pathologist_1
Pathologist_2
Pathologist_3	120
120
120	3.4500000
3.8833333
3.0333333	0.7316380
0.4335380
0.9867469	2.0000000
1.0000000
0	4.0000000
4.0000000
4.0000000	

N	Mean	Std Dev	Std Err	Minimum	Maximum	
120	-0.2500	0.9190	0.0839	-2.0000	2.0000	


Mean	95% CL Mean	Std Dev	95% CL Std Dev	
-0.2500	-Infty	-0.1109	0.9190	0.8156	1.0526	


DF	t Value	Pr < t	
119	-2.98	0.0017	


N	Mean	Std Dev	Std Err	Minimum	Maximum	
120	-0.0583	0.6648	0.0607	-3.0000	2.0000	


Mean	95% CL Mean	Std Dev	95% CL Std Dev	
-0.0583	-Infty	0.0423	0.6648	0.5900	0.7615	


DF	t Value	Pr < t	
119	-0.96	0.1692	


N	Mean	Std Dev	Std Err	Minimum	Maximum	
120	-0.4500	1.1291	0.1031	-3.0000	3.0000	


Mean	95% CL Mean	Std Dev	95% CL Std Dev	
-0.4500	-Infty	-0.2791	1.1291	1.0020	1.2933	


DF	t Value	Pr < t	
119	-4.37	<.0001	


target_output	N Obs	Variable	N	Mean	Std Dev	Minimum	Maximum	
output	120	Pathologist_1
Pathologist_2
Pathologist_3	120
120
120	3.6083333
3.6583333
3.9833333	0.5391533
0.7503034
0.1285559	2.0000000
1.0000000
3.0000000	4.0000000
4.0000000
4.0000000	
target	120	Pathologist_1
Pathologist_2
Pathologist_3	120
120
120	3.6333333
3.8416667
3.9833333	0.5489996
0.4490107
0.1825742	2.0000000
2.0000000
2.0000000	4.0000000
4.0000000
4.0000000	

N	Mean	Std Dev	Std Err	Minimum	Maximum	
120	-0.0250	0.7272	0.0664	-2.0000	2.0000	


Mean	95% CL Mean	Std Dev	95% CL Std Dev	
-0.0250	-Infty	0.0850	0.7272	0.6454	0.8329	


DF	t Value	Pr < t	
119	-0.38	0.3536	


N	Mean	Std Dev	Std Err	Minimum	Maximum	
120	-0.1833	0.8695	0.0794	-3.0000	2.0000	


Mean	95% CL Mean	Std Dev	95% CL Std Dev	
-0.1833	-Infty	-0.0518	0.8695	0.7717	0.9960	


DF	t Value	Pr < t	
119	-2.31	0.0113	


N	Mean	Std Dev	Std Err	Minimum	Maximum	
120	0	0.2245	0.0205	-1.0000	2.0000	


Mean	95% CL Mean	Std Dev	95% CL Std Dev	
0	-Infty	0.0340	0.2245	0.1993	0.2572	


DF	t Value	Pr < t	
119	0.00	0.5000	


target_output	N Obs	Variable	N	Mean	Std Dev	Minimum	Maximum	
output	120	Pathologist_1
Pathologist_2
Pathologist_3	120
120
120	2.7833333
3.8750000
2.7500000	0.4882370
0.3793515
0.6514367	2.0000000
2.0000000
2.0000000	4.0000000
4.0000000
4.0000000	
target	120	Pathologist_1
Pathologist_2
Pathologist_3	120
120
120	2.9833333
3.9000000
2.8583333	0.5497644
0.3757346
0.7135641	2.0000000
2.0000000
1.0000000	4.0000000
4.0000000
4.0000000	

N	Mean	Std Dev	Std Err	Minimum	Maximum	
120	-0.2000	0.6686	0.0610	-2.0000	1.0000	


Mean	95% CL Mean	Std Dev	95% CL Std Dev	
-0.2000	-Infty	-0.0988	0.6686	0.5934	0.7659	


DF	t Value	Pr < t	
119	-3.28	0.0007	


N	Mean	Std Dev	Std Err	Minimum	Maximum	
120	-0.0250	0.5260	0.0480	-2.0000	2.0000	


Mean	95% CL Mean	Std Dev	95% CL Std Dev	
-0.0250	-Infty	0.0546	0.5260	0.4668	0.6025	


DF	t Value	Pr < t	
119	-0.52	0.3018	


N	Mean	Std Dev	Std Err	Minimum	Maximum	
120	-0.1083	0.8280	0.0756	-2.0000	2.0000	


Mean	95% CL Mean	Std Dev	95% CL Std Dev	
-0.1083	-Infty	0.0170	0.8280	0.7349	0.9485	


DF	t Value	Pr < t	
119	-1.43	0.0772	
