# Supplementary material for: Label-Free Virtual HER2 Immunohistochemical Staining of Breast Tissue using Deep Learning
Source: BME Front. 2022 Oct 25;2022:9786242. doi: 10.34133/2022/9786242 (PMC10521710; doi:10.34133/2022/9786242)
Supplement: Supplementary Materials — The Supplementary Materials include Supplementary Figures 1-9, Supplementary Tables 1-2 and Supplementary Note 1 (IHC HER2 staining protocol). [file 9786242.f1.zip › Supplementary_Info_HER2_VirtualStaining_vF TBS Rev vFinal.pdf]

Supplementary information:

Label-free virtual HER2 immunohistochemical staining of breast tissue using deep learning

Bijie Bai [†,1,2,3], Hongda Wang[†,1,2,3], Yuzhu Li[†,1,2,3], Kevin de Haan[1,2,3], Francesco Colonnese[4], Yujie Wan[5], Jingyi Zuo[4], Ngan B. Doan[6], Xiaoran Zhang[1], Yijie Zhang[1,2,3], Jingxi Li[1,2,3], Xilin Yang[1,2,3], Wenjie Dong[7], Morgan Angus Darrow[8], Elham Kamangar[8], Han Sung Lee[8], Yair Rivenson[1,2,3], and Aydogan Ozcan[*,1,2,3,9]

[1]Electrical and Computer Engineering Department, University of California, Los Angeles, CA, 90095, USA.

[2]Bioengineering Department, University of California, Los Angeles, 90095, USA.

[3]California NanoSystems Institute (CNSI), University of California, Los Angeles, CA, USA.

[4]Computer Science Department, University of California, Los Angeles, CA, USA.

[5]Physics and Astronomy Department, University of California, Los Angeles, CA, 90095, USA

[6]Translational Pathology Core Laboratory, University of California, Los Angeles, CA, 90095, USA

[7] Statistics Department, University of California, Los Angeles, CA, 90095, USA.

[8]Department of Pathology and Laboratory Medicine, University of California at Davis, Sacramento, CA, 95817, USA.

[9]Department of Surgery, University of California, Los Angeles, CA, 90095, USA

[†]Equal contributing authors

[*]Correspondence: Aydogan Ozcan. Email: ozcan@ucla.edu

Supplementary Figure 1. **Examples of unsuccessful chemical IHC staining. a,** The pseudo-colored autofluorescence image captured using an unlabeled breast tissue section. **b,** Virtual HER2 staining predicted by our generator network. **c,** The same tissue section suffered from severe tissue damage and loss during standard IHC HER2 staining. **d,** The IHC staining of a serially sliced section from the same sample block. **e,** Pseudo-colored autofluorescence image captured using another unlabeled breast tissue section. **f,** Virtual HER2 staining predicted by our generator network. **g,** The same tissue section experienced false negative IHC HER2 staining (i.e., unsuccessful IHC staining). **h,** The IHC staining of a serially sliced section from the same sample block.

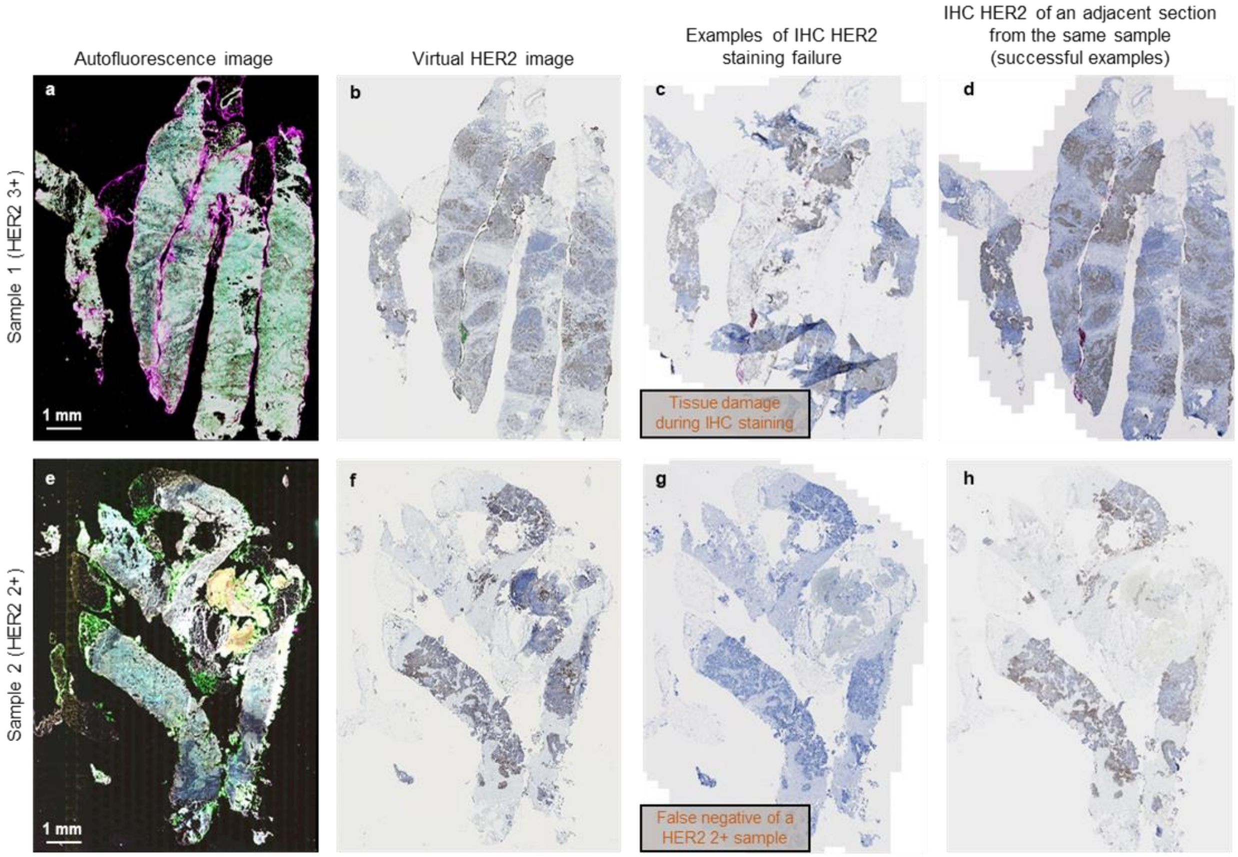

Supplementary Figure 2. HER2 scores corresponding to image patches. a, Histograms of HER2 scores graded based on the image patches cropped from virtual HER2 WSI (blue curves) and standard IHC HER2 WSI (orange curves) of each patient. **b,** Individual HER2 scores corresponding to image patches graded by three pathologists.

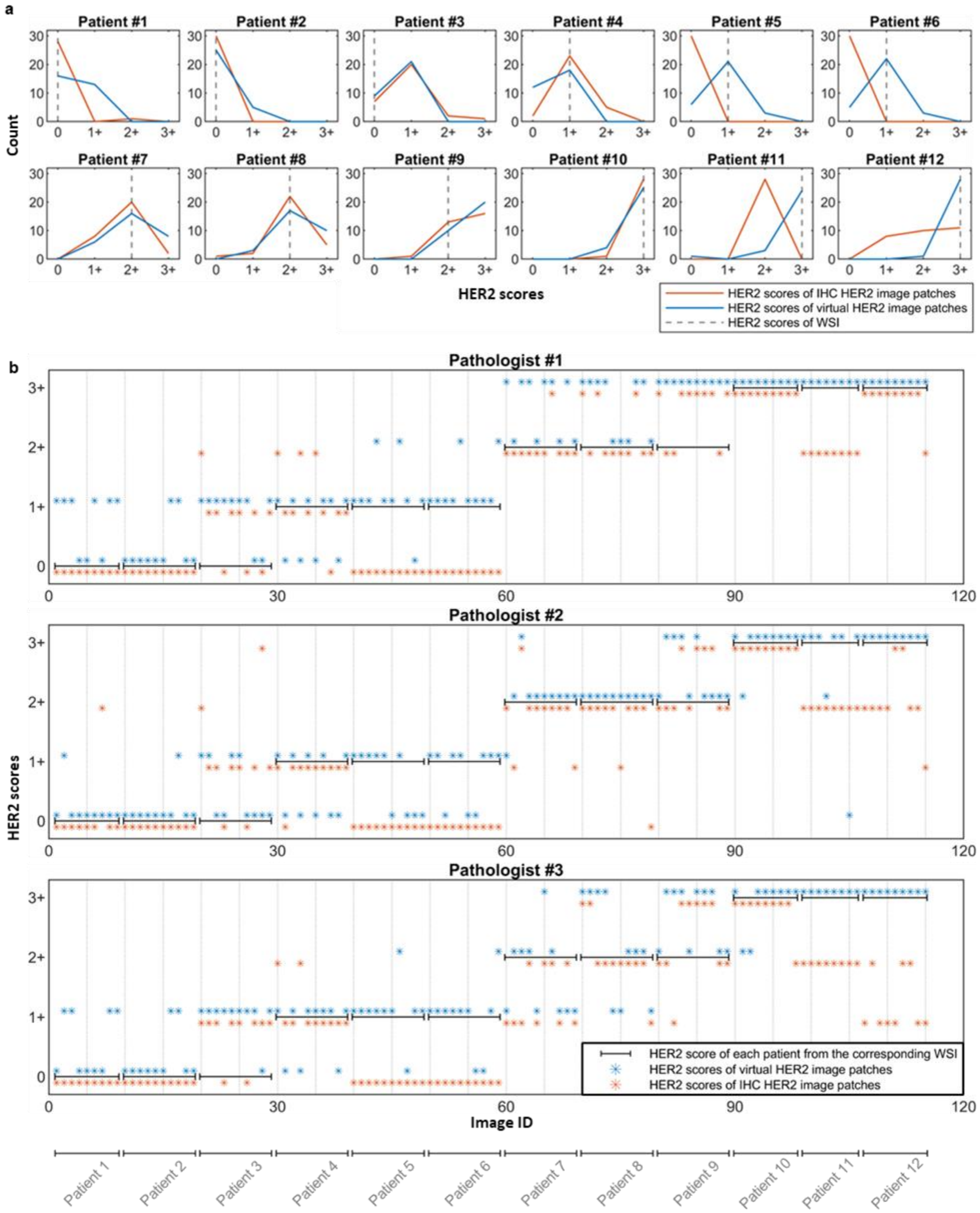

Supplementary Figure 3. **Comparison of virtual staining network performance with different autofluorescence input channels. a,** Visual comparisons of virtual staining networks trained with one (DAPI), two (DAPI + FITC), three (DAPI + FITC + TxRed), and four (DAPI + FITC + TxRed + Cy5) autofluorescence input channels, showing the improved results as the number of input channels increases. **b,** Quantitative evaluations of virtual staining networks trained with different numbers of autofluorescence input channels. MSE, SSIM, and SSIM of membrane color channel (i.e., DAB stain) were calculated using the network output and the ground truth images.

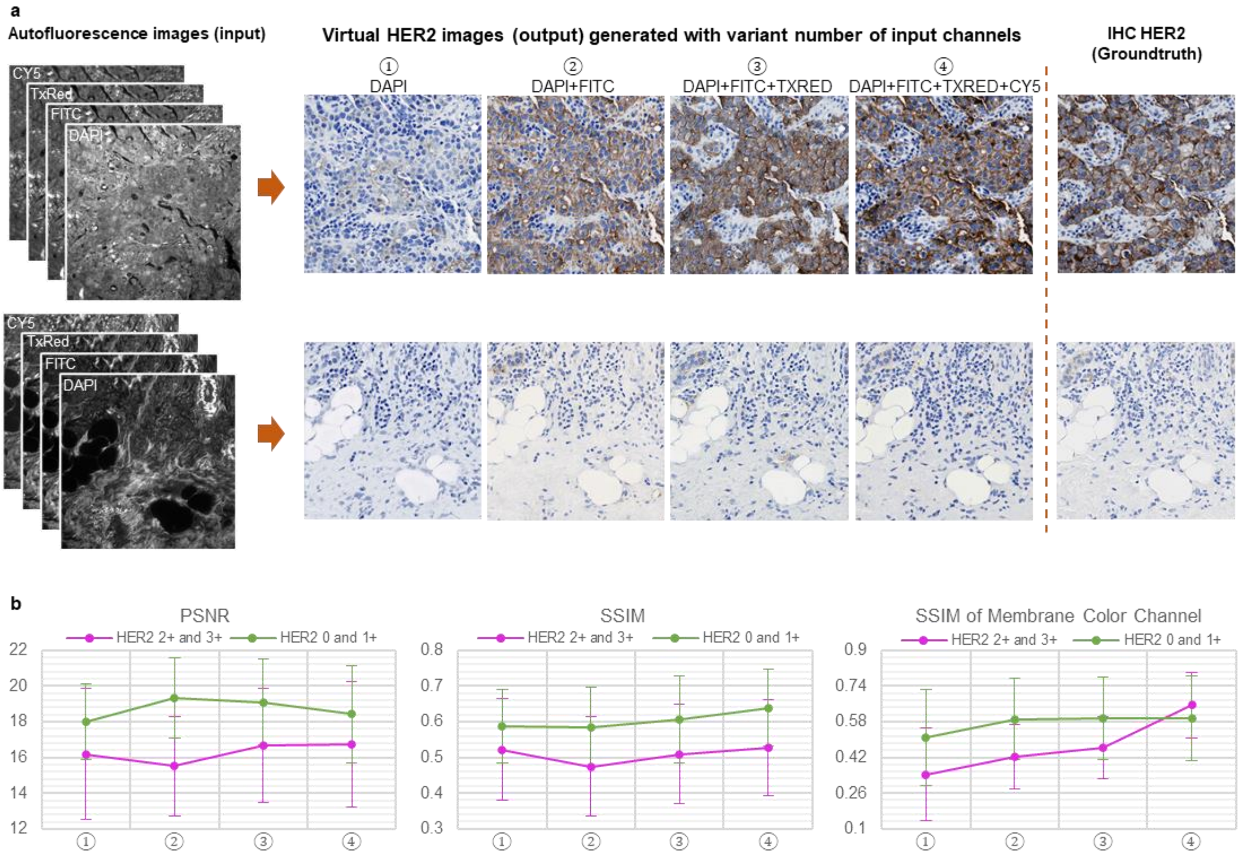

Supplementary Figure 4. **Examples of color deconvolution to split the diaminobenzidine (DAB) stain channel and the Hematoxylin stain channel. a,** Color deconvolution of a HER2 positive region. **b,** Color deconvolution of a HER2 negative region.

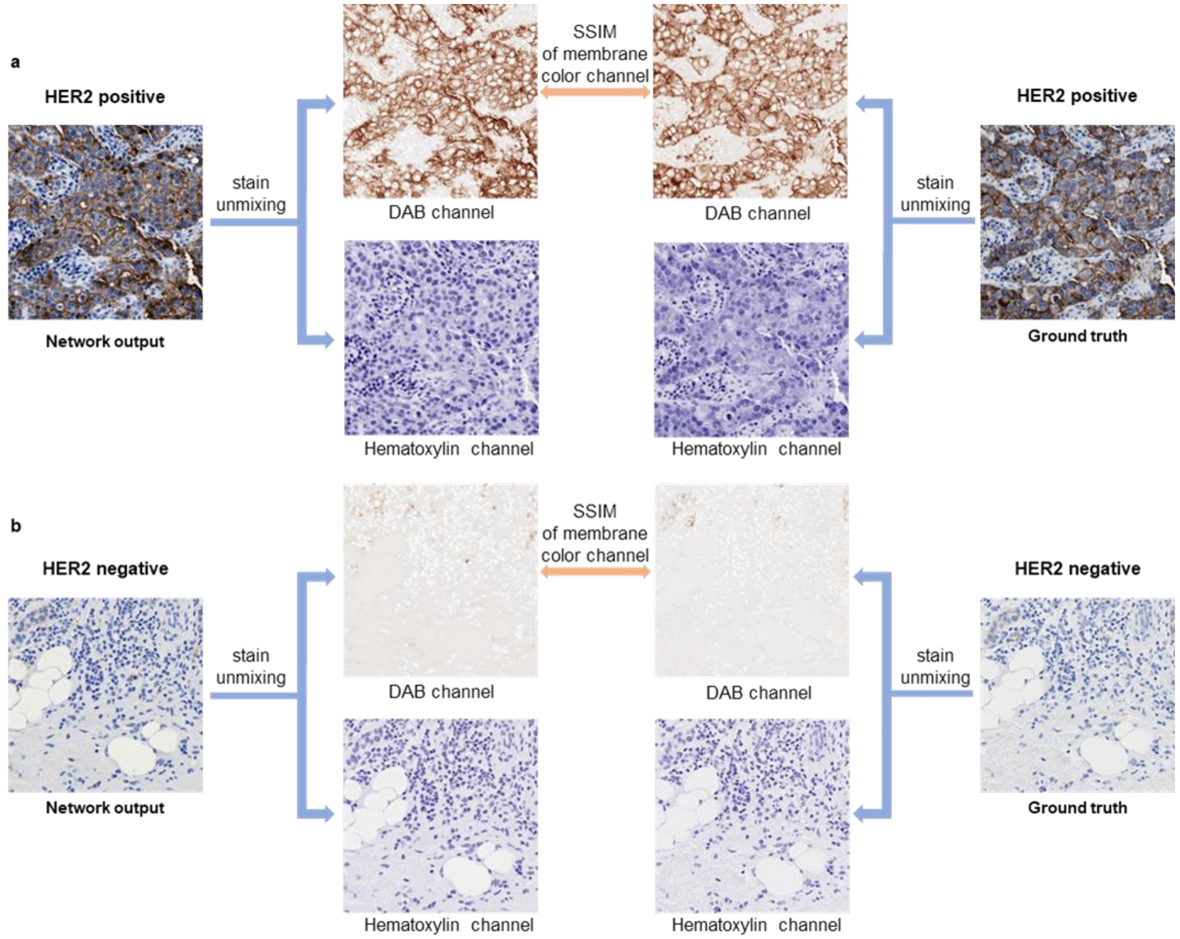

Supplementary Figure 5. **Quantitative comparison of different virtual staining network architectures.** Both the visual and numerical comparisons revealed the superior performance of the attention-gated GAN used in our work compared to other network architectures.

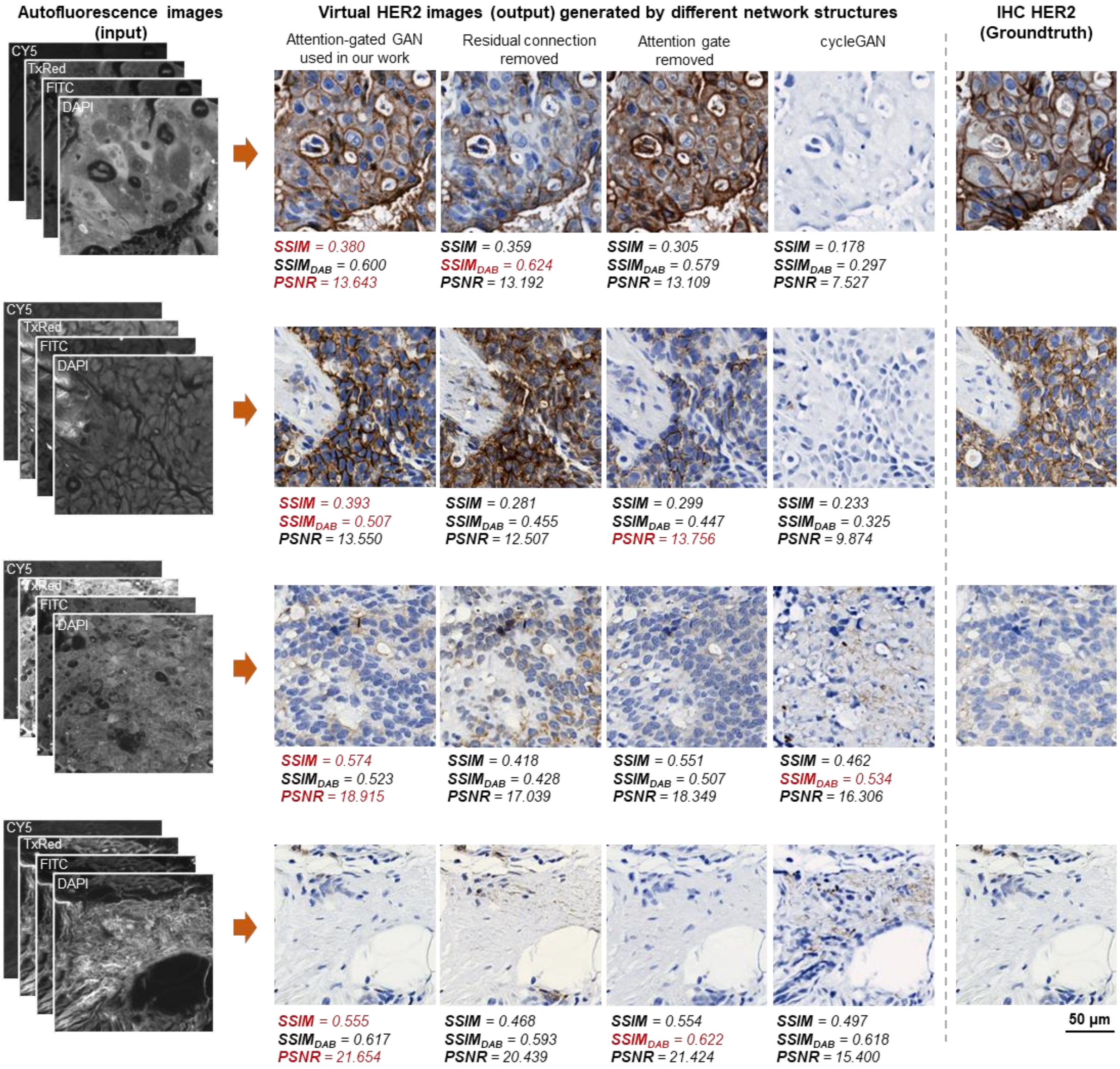

Supplementary Figure 6. **Comparison of the color distributions of the output images (with strong HER2 expression) generated by different virtual staining networks.** The color distributions of the output images generated by the attention-gated GAN closely match the color distributions of the standard IHC ground truth images.

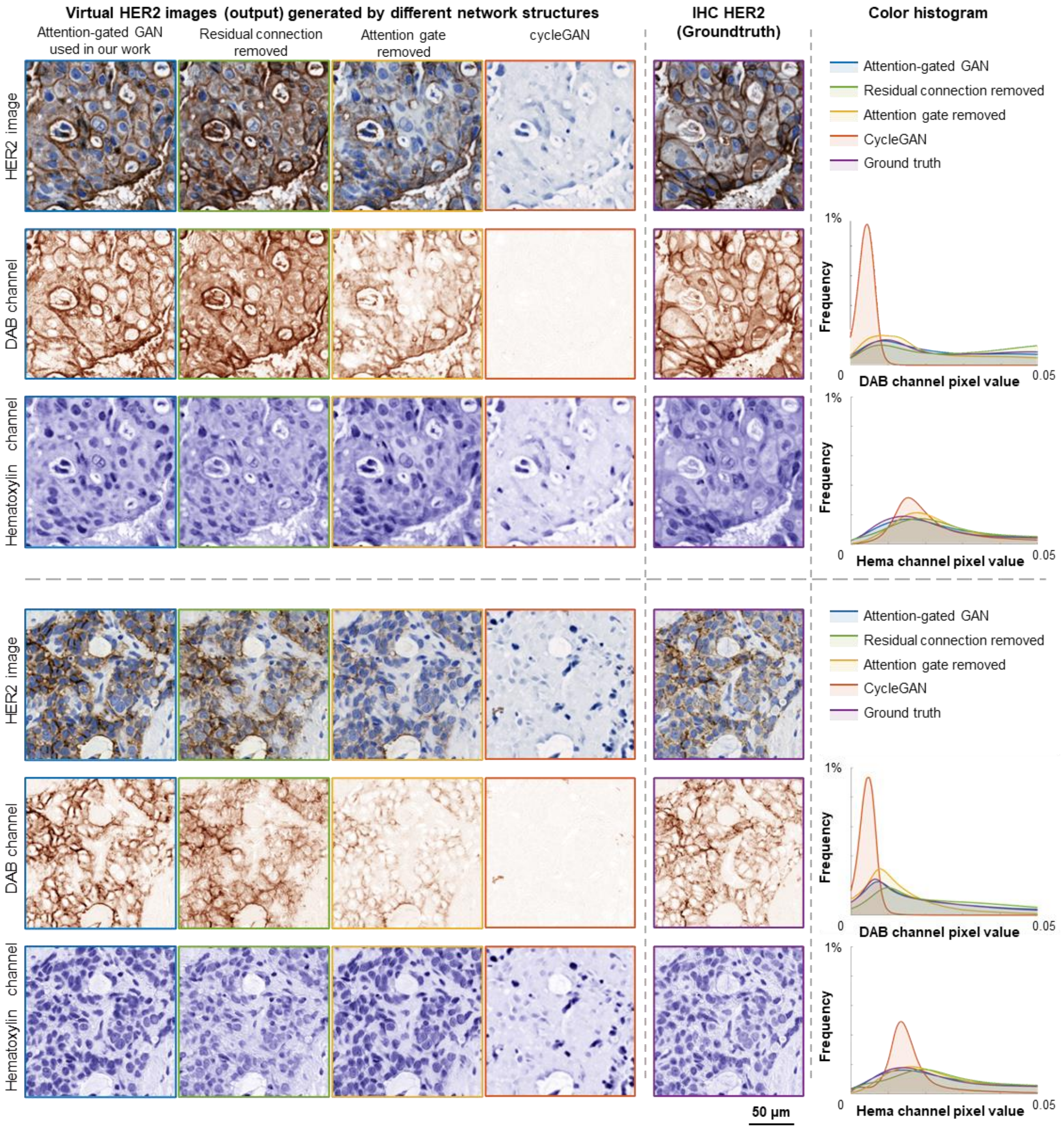

Supplementary Figure 7. **Comparison of the color distributions of the output images (with weak HER2 expression) generated by different virtual staining networks.** The color distributions of the output images generated by the attention-gated GAN closely match the color distributions of the standard IHC ground truth images.

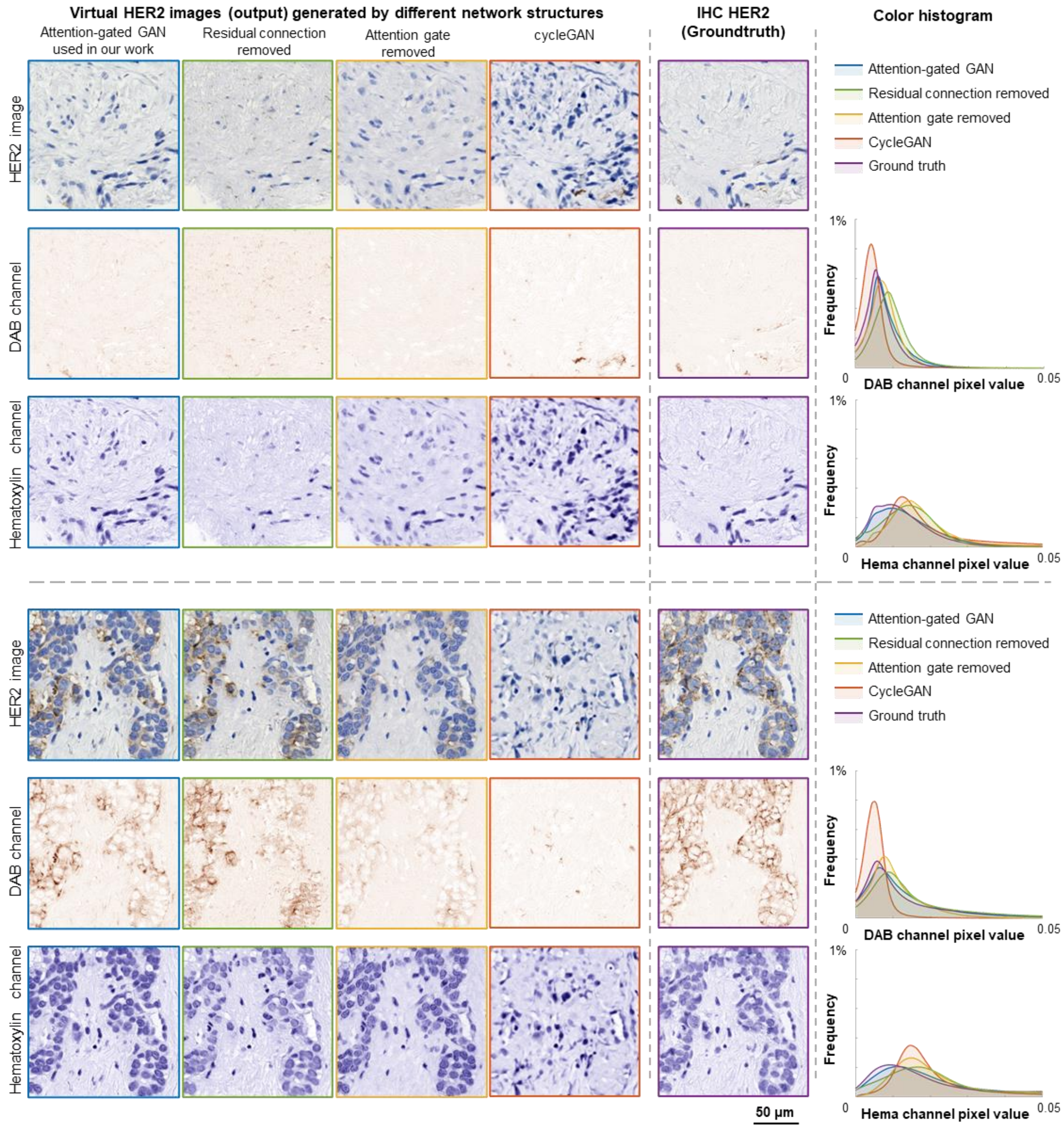

Supplementary Figure 8. **Image preprocessing and registration workflow. a,** Stitched autofluorescence WSI (before the IHC staining) and the bright-field WSI (after the IHC staining) of the same tissue section. **b,** Global registration of autofluorescence WSI and bright-field WSI by detecting and matching the SURF feature points. **c,** Cropped coarsely matched autofluorescence and bright-field image tiles. **d,** Registration model was trained to transform the autofluorescence images to the bright-field images. **e,** Registration model output and ground truth images. **f,** The ground truth images were registered to autofluorescence images using an elastic registration algorithm. **g,** Perfectly matched autofluorescence and bright-field image patches were obtained after 3-5 rounds of iterative registration.

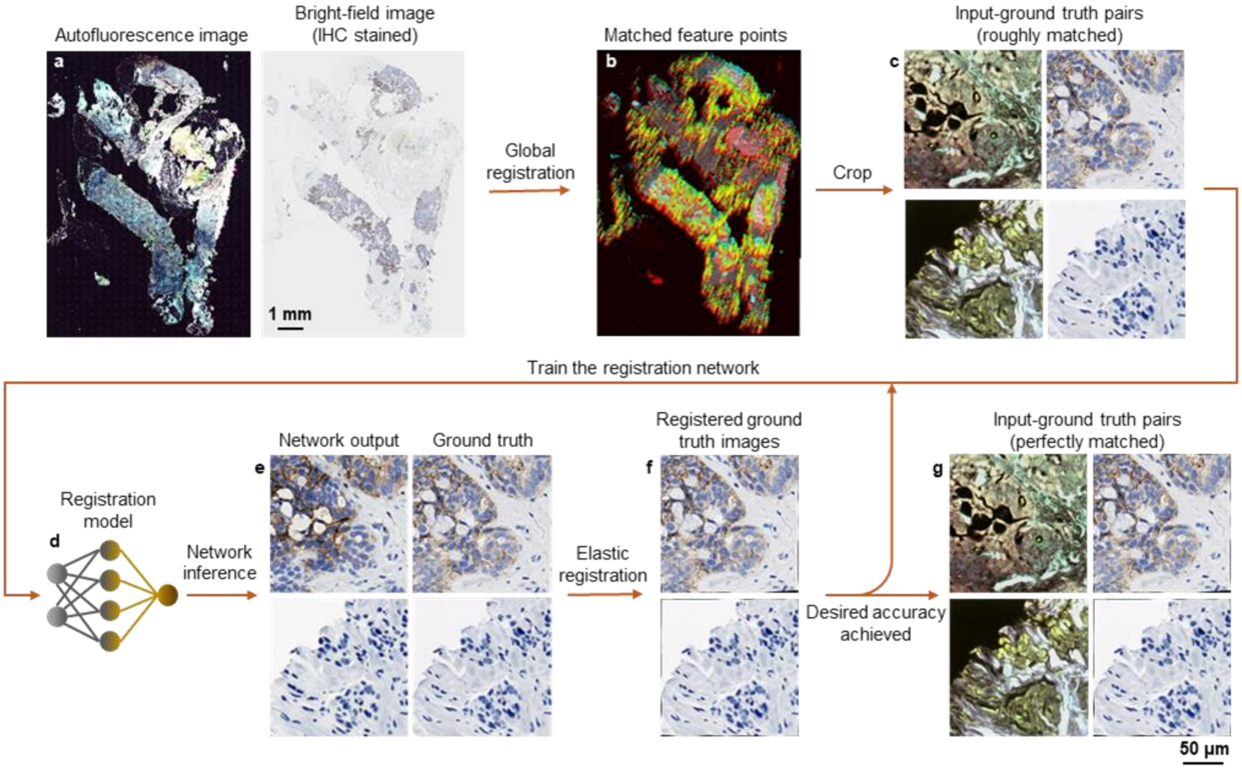

Supplementary Figure 9. Extraction of the nucleus and membrane stain features based on color deconvolution and segmentation algorithms.

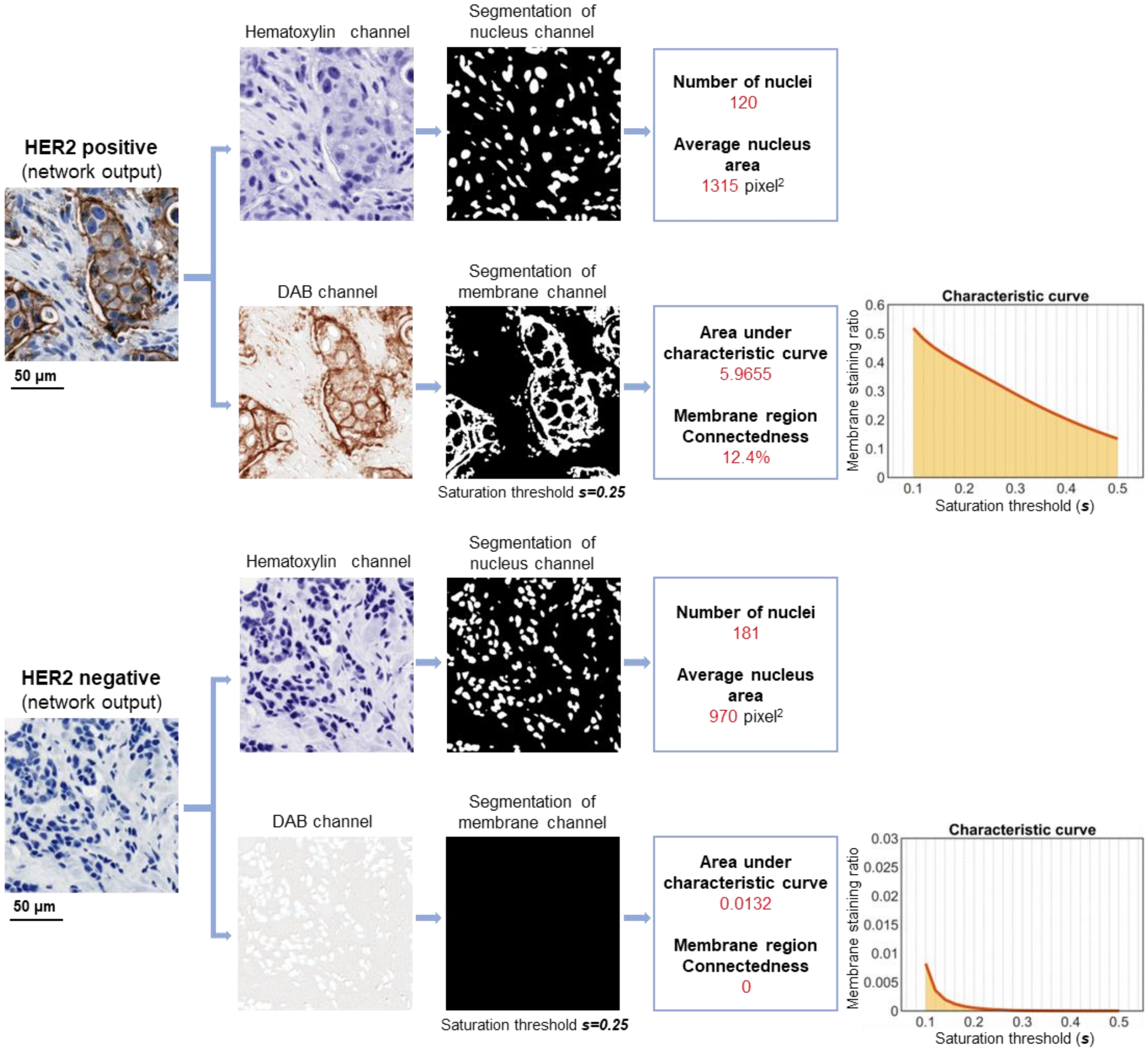

Supplementary Table 1. **Chi-Square test table for HER2 status assessment.**

	Virtual HER2	***IHC HER2***	***Total***
Disagree	14<br>**38.89**	17<br>**47.22**	31
Agree	22<br>**61.11**	19<br>**52.78**	41
Total	36	36	72

Statistic	***DF***	***Value***	***Prob.***
Chi-Square	1	0.5098	0.4752
Likelihood Ratio Chi-Square	1	0.5105	0.4749
Continuity Adj. Chi-Square	1	0.2266	0.6341
Mantel-Haenszel Chi-Square	1	0.5028	0.4783

Supplementary Table 2. **Summary of t-test results for staining quality assessment.**

Difference = quality score of virtually stained image – quality score of IHC stained image

Null hypothesis: $Difference_{Virtual-IHC} \geq 0$

Alternative hypothesis: $Difference_{Virtual-IHC} < 0$

	Pathologist #1		***Pathologist #2***		***Pathologist #3***	
	Mean	***Pr < t***	***Mean***	***Pr < t***	***Mean***	***Pr < t***
Membrane clearness	0.1000	0.8615	-0.1207	0.1986	-0.1000	0.2324
Absence of staining artifacts	-0.0250	0.3536	-0.1833	0.0113	0.0000	0.5000
Absence of excessive background staining	-0.2500	0.0017	-0.0583	0.1692	-0.4500	<.0001
Nuclear details	-0.2000	0.0007	-0.0250	0.3018	-0.1083	0.0772

Supplementary Note 1. IHC HER2 staining protocol

Paraffin-embedded sections were cut at 4μm thickness and paraffin was removed with xylene and rehydrated through graded ethanol. Endogenous peroxidase activity was blocked with 3% hydrogen peroxide in methanol for 10 min. Heat-induced antigen retrieval (HIER) was carried out for all sections in AR9 buffer (AR9001KT Akoya) using a decloaking chamber (Biocare Medical) at 95°C for 25 min. The slides were then stained with HER2 antibody (cell signaling, 4290, 1-200) at 4°C overnight. The signal was detected using the DakoCytomation Envision System Labelled Polymer HRP anti-rabbit (Agilent K4003, ready to use). All sections were visualized with the diaminobenzidine reaction and counterstained with hematoxylin.
